# Looking ahead at the potential benefits of biotechnology-derived allergen therapeutics

**DOI:** 10.1186/1476-7961-5-3

**Published:** 2007-07-04

**Authors:** Jason Behrmann

**Affiliations:** 1 Programmes de bioéthique, Université de Montréal, C.P. 6128, succursale centre-ville, Montréal, Québec, H3C 3J7, Canada

## Abstract

While biotechnology-derived allergen therapeutics show promise in improving the safety of immunotherapy, they may prove to have additional benefits in comparison to conventional allergenic extracts that deserve commentary. These issues range from product stability and compatibility to medical practice issues, which will be the focus of this article.

## Background

Allergies, or hyperimmune responses to ordinary entities in our environment, are extremely common chronic pathological conditions that affect 10 to 25% of the population [1,2] and have significant impacts on productivity, quality of life issues, and costs towards the administration of health services [3]. Common practices for ameliorating allergy symptoms include allergen avoidance, allergen elimination, pharmacotherapy (such as the administration of anti-histamines), and allergen-specific immunotherapy. Allergen-specific immunotherapy (SIT) was developed in 1911 [4] and is the only treatment that attends to the root cause, rather than the clinical symptoms, of allergic reactions. The most common method of SIT employs the subcutaneous injection of extracts of biological material that contains the allergen. Gradual, increasing doses of the administered allergenic extract serves to physiologically moderate the immune system so that it is less primed for a hyperimmune response upon subsequent exposure to the allergen (for a concise review, see: [5], p. 49–51). An inherent problem with SIT is that it carries a low but significant risk of inducing severe and sometimes fatal systemic reactions such as anaphylaxis [6]. It is therefore advised that SIT be performed by trained allergists in facilities that are equipped to treat anaphylactic reactions, and that patients be monitored for 30 minutes after the treatment [5].

After decades of using biologic extracts for SIT, technology has progressed to the point where the corresponding genes for key allergens have been identified and cloned, making possible the production (and strategic modification) of recombinant allergens via biotechnological techniques [Allergen products [7]]. Of particular interest is the potential, noted in several reviews, for the development of novel drugs that can minimize the possibility of adverse reactions to immunotherapy [8,9]. Added to the fact that recombinant allergens could be purified to near homogeneity – an improvement to current extracts that are complex mixtures containing largely non-allergenic and uncharacterized material – recombinant allergens are being engineered to have reduced IgE immunoglobulin binding capacity while retaining their therapeutic attributes for immunotherapy. This essentially means that future immunotherapy might be performed with 'hypo-allergenic' allergens that pose little risk for anaphylactic reactions.

With the possibility of batch production of homogenous allergen proteins via biotechnological techniques, the opportunity to increase the therapeutic efficacy of allergen vaccines can surface through conjugation of the purified allergen to immunostimulatory DNA moieties [reviewed in: [10]]. Unmethylated CG dinucleotide DNA sequences, found in certain bacterial species, possess immunostimulatory capacities [11]. Interestingly, conjugation of synthetic versions of these DNA sequences to major short ragweed allergen (Amb a 1) has been shown to enhance the immunogenicity, while lowering the allegenicity, of the protein [12]. Accumulative research is dissecting the mechanism behind the improved therapeutic efficacy of immunostimulatory DNA-conjugated allergens (ISD-allergens). In brief, the DNA conjugates appear to stimulate the immune system so that the development of a Th1-type immune response (immunotherapeutic IgG antibody production) is favoured over a Th2-type response (IgE and inflammatory cytokine production) [10]. Preliminary studies demonstrate that the allergenicity of ISD-allergen products have the potential to be several folds less allergenic than conventional allergenic extracts [10,13]. These observations suggest that vaccines of allergens conjugated to immunostimulatory DNA moieties could ameliorate the safety and efficacy of future SIT regimes.

Further biotechnological developments raise the possibility that proteinaceous vaccines for allergen-specific immunotherapy may one day combat allergies alongside plasmid-DNA (pDNA) vaccines [14,15]. Instead of injecting allergenic material, bacterially-produced plasmids encoding for the allergenic entity could be used to produce the allergen endogenously once transfected into the recipient's cells. Though still in the early stages of development (thus the clinical efficacy of these drugs is highly debateable), preliminary results from mouse models show that pDNA vaccines may be suitable for SIT and other forms of immunization [16-18]. Furthermore, a study by McConkey and colleagues [19] demonstrated that immunization with plasmid vaccines in conjunction with proteinaceous vaccines greatly augmented the immune response in comparison to vaccination with the proteinaceous vaccine alone. An interesting attribute of the pDNA vaccine is that since the allergen is produced endogenously, it is presented to the immune system at a very low concentration over a prolonged period. The slow production of allergen makes it virtually impossible for a severe hyperimmune response to occur, as can be the case with conventional allergenic extracts. Thus, life-threatening systemic reactions appear unlikely with pDNA vaccines [17], which would further increase the safety and utility of SIT.

Biotechnology has the potential for the development of novel drugs for the treatment of allergies that may have several attributes that are distinct from current therapeutics. While these innovations are known to differ at the molecular and pharmacological level, they may have broader implications related to medical practice and protocols for the treatment of allergies that are less predictable.

## Discussion

### Stability and compatibility of therapeutics

An unwanted attribute of some allergenic extracts is that the final product may contain proteolytic enzymes. This is observed particularly in extracts made from biologic material of dust mites, cat and dog pelts, and some pollen varieties [20]. Endogenous protease activity is problematic because it can degrade active ingredients in the therapeutic, resulting in reduced product stability and shelf-life [21]. Stability can be ameliorated by formulating the extract in glycerol [22], but these products are not favoured since glycerol produces pain at the injection site [5]. Moreover, since many patients undergoing SIT are allergic to more than one allergen, the mixing of allergenic extracts is often required to include all of the relevant allergens [5]. The mixing of proteolytic extracts with others must be avoided – usually by isolating specific extracts in distinct vials – but the therapeutic regime will require additional injections for the distinct extracts, which can make the therapy less pleasant for the patient. Deciding what extracts are compatible with each other may also prove difficult as the potential for proteolytic activity in an extract is not marked on the product label. However, problems associated with proteolytic degradation could be circumvented with recombinant allergens, which 1) would not be contaminated with unwanted proteolytic enzymes, and 2) could be genetically engineered to be free of proteolytic function. In summary, recombinant allergen products formulated to be free of proteolytic activity may be of increased stability without the need for glycerol and may be easier to formulate as mixtures for individualized therapeutic regimes.

### Minimizing localized and systemic reactions: broader implications

The ramifications of minimizing localized and systemic reactions, especially anaphylaxis, through the application of less allergenic varieties of recombinant allergens and ISD-allergens, or pDNA vaccines, could be far reaching. Current allergenic extracts have a high propensity to produce localized reactions such as itchiness and swelling at the injection site. For most patients, this is a mild annoyance, but this can be a significant psychological factor when administering SIT to young children [23]. Less allergenic recombinant varieties or pDNA versions could eliminate localized reactions altogether, while also removing the need for the 30 minute precautionary wait time currently necessary to monitor for anaphylaxis. This in turn would free-up time and space within allergy clinics, allowing for more prompt treatment of additional patients and could produce monetary savings to the healthcare system. Additionally, the shorter time within the allergy clinic in this situation may improve patient compliance with the therapeutic regime. The wait time is viewed by many patients, as observed by experts [24], as a displeasure and motivates some to terminate the therapeutic regime prematurely.

### Expanding treatment options

The incidence of allergies within the general population is increasing in prevalence [25,26], and will necessitate broader access to appropriate therapies. Such a demand will be problematic without sufficient numbers of allergy specialists, especially in remote areas. The potentially increased safety of biotechnology-derived allergen therapeutics raises the real possibility of being able to administer therapeutic regimes through general health care facilities that need not be equipped to treat anaphylactic reactions. This would allow much broader access to SIT and permit allergists to focus their resources on the diagnosis and evaluation of therapeutic needs for patients. As an aside, the American College of Allergy, Asthma & Immunology [27] has noted that managed care practices for health services in the U.S. have encouraged the administration of immunotherapy by primary care physicians. Biotechnology-derived allergen therapeutics would be a more appropriate (and safer) product for primary care physicians than current SIT allergen products, and likely improve patient compliance.

Recombinant and ISD-allergens may make certain risky but effective SIT protocols more acceptable, as in the case of rush immunotherapy. In rush immunotherapy, large doses of an allergenic extract are administered over a very short period of time, such that the maximum dose level of the therapeutic is reached within hours to days, instead of weeks or months for conventional SIT [24]. While greatly increasing efficiency (and also patient compliance), rush immunotherapy is burdened with a significantly higher incidence of adverse reactions (18 times more prevalent than with conventional SIT [5]). Were rush protocols to be performed with less allergenic recombinant or ISD-allergens, one could foresee the benefits of expedited immunotherapy and compliance without the associated safety concerns.

## Conclusion

While the science of biotechnology-derived allergy therapeutics is attractive and has drawn recent attention, the potential benefits of these therapeutics for improving therapeutic protocols, patient compliance, and broader administration of immunotherapy is equally attractive. At the moment, the potential benefits of biotechnology in the field of allergy treatment can only be envisioned, and whether these benefits will indeed manifest still requires many years of research – the results from clinical trials of biotechnology-derived allergen therapeutics are eagerly awaited and the commercialization of a variety of these products appears likely [28]. Thus, it appears that after decades of using crude extracts of biological material for SIT, the realm of allergy therapeutics is positioned to enter the biotech sphere that has proven its revolutionary potential in numerous fields ranging from oncology and HIV therapeutics to vaccines [29].

## Competing interests

The author(s) declare that they have no competing interests.
